# Posterior cerebral artery stroke by reverse flow embolism in thoracic outlet syndrome - a case report

**DOI:** 10.1186/s12883-020-01797-y

**Published:** 2020-06-04

**Authors:** Adam Celier, Simon Chabay, Aurélien Maurizot, Frédéric Cochennec, Daniela Stanciu, Fernando Pico

**Affiliations:** 1 Department of neurology and stroke center, Versailles Mignot Hospital, 78150 Le Chesnay, France; 2 Department of angiology, Versailles Mignot Hospital, 78150 Le Chesnay, France; 3 Department of vascular surgery, Henri Mondor University Hospital, 94000 Créteil, France; 4 Versailles Saint Quentin en Yvelines and Paris Saclay University, 78000 Versailles, France

**Keywords:** Arterial thoracic outlet syndrome, Ischemic stroke, Reverse flow embolism, Ultrasound

## Abstract

**Background:**

Arterial thoracic outlet syndrome is a rare condition characterized by a subclavian artery pathology associated with a bone abnormality. It is rarely associated with thromboembolic stroke. The mechanism of cerebral embolism associated with thoracic outlet syndrome have rarely been demonstrated. We present here a fully studied case with a high probability of reverse flow embolism.

**Case presentation:**

A 24-year-old man with a known arterial thoracic outlet syndrome presented with a right cerebral posterior artery brain infarction. An ultrasound examination depicted the compression of the right subclavian artery in the scalene defile with a post stenotic aneurysm and the presence of a floating thrombus in this aneurysm. There was a reverse flow during diastole in this aneurysm. Anticoagulation was carried out with the disappearance of the floating thrombus with no new clinical or brain MRI event. Corrective surgery of this thoracic outlet syndrome was performed one month after stroke.

**Conclusion:**

Very few cases of stroke in arterial thoracic outlet syndrome have been described with thorough dynamic vascular imaging. To our knowledge, this is the fourth reported case that advocates for a reverse flow embolism mechanism in stroke associated with thoracic outlet syndrome, and the first to realize an extensive ultrasound and doppler workup.

## Background

Thoracic outlet syndrome (TOS) is a common condition and results of the compression of the neurovascular structures in the thoracic outlet, defined as the interval from the supraclavicular fossa to the axilla that passes between the clavicle and the first rib [1]. The thoracic outlet consists of three compartments: the interscalene triangle, the costoclavicular space, and the subcoracoid space [2]. The interscalene triangle is composed of two muscles, the anterior and middle scalenes, and the first rib. The subclavian artery and the brachial plexus pass through the interscalene triangle, whereas the subclavian vein courses anteriorly to it [1]. But all three structures course through the costoclavicular space, located between the clavicle, the subclavius muscle, the anterior scalene and the first rib. Finally, the brachial plexus passes through the subcoracoid space, bordered by the pectoralis minor muscle and the ribs, and the subclavian artery and vein course through it as the axillary artery and vein [1]. The subclavius muscle can greatly reduce the caliber of the canal. It includes brachial plexus compression (neurogenic TOS), subclavian vein thrombosis (venous TOS), and subclavian artery (SCA) compression (arterial TOS). Neurogenic TOS is by far the most frequent form (90–95%) [3], followed by venous TOS (2–5%) [3]. Arterial TOS is the least frequent form (1–2%) [3], but its clinical presentation is distinguished by the potential life- and limb-threatening thromboembolic complications. It most often anatomically concerns the interscalene triangle, and is frequently associated to a cervical rib [4]. This condition can be found in approximately 1% of the population and is bilateral in 50% of the cases. Women are two times more likely to be affected by a cervical rib [3].

Cerebral embolism is a rare but known complication of arterial TOS [4–16]. Two mechanisms are proposed (in case of native arteries without chronic occlusion of the SCA): either retrograde spreading of a thrombus or backward embolism within the SCA [4].

We report a case of right posterior cerebral artery (PCA) ischemic stroke in a young adult known for having an arterial TOS. The findings of ultrasound and doppler examination of supra-aortic arteries suggest a high probability of reverse flow embolism mechanism in this case.

## Case presentation

A 24-year-old male presented in August 2017 with transient memory loss and paresthesia of the left lower limb. The patient was right handed with no tobacco intoxication and occasional alcohol intake. The patient’s main medical history was a known right arterial thoracic outlet syndrome diagnosed in Nov 2016 after a 3 days episode of cold and painful right hand during the night, and Raynaud’s syndrome when he was playing table tennis.

**Fig. 1:**
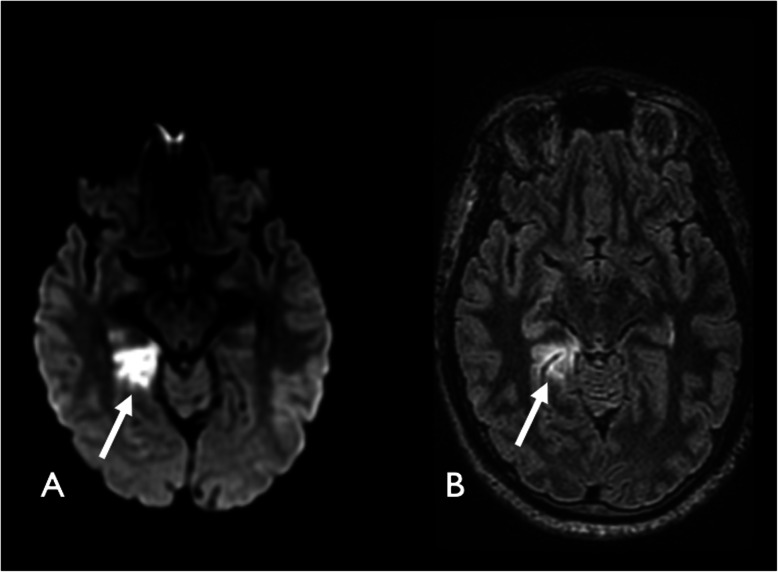
Axial diffusion (**a**) and FLAIR (**b**) MRI showing acute right posterior cerebral artery infarction (arrows)

**Fig. 2:**
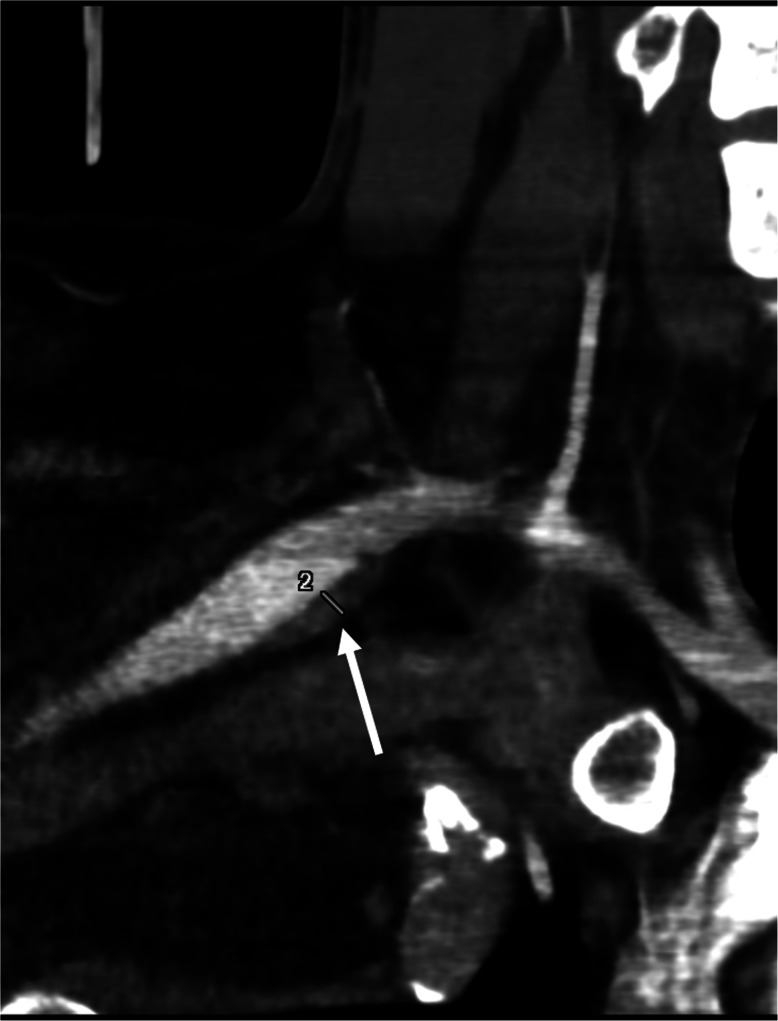
CT angiography showing a 24.1 mm thrombus (arrow) in the subclavian artery

He underwent in January 2017 a CT angiography of the supra-aortic arteries that showed an extrinsic compression of the right subclavian artery with mild stenosis of the retro-scalar subclavian artery, associated to a typical post-stenotic dilatation with no thrombus inside. Ultrasound examination of the supra-aortic arteries depicted a focal occlusion of the termination of the right humeral artery. Upper limb electroneuromyography was normal. Cervical spine and chest X ray did not show a cervical rib. Cervical CT scan showed anterior synostosis of the 1st and 2nd rib. Due to the occlusion of the right humeral artery and the post-stenotic dilatation on the right subclavian artery, it was decided to plan a surgery of the first rib associated with an arterial bridging for Sept 2017. On the day before admission in our unit in August 2017, the patient had an episode of transient memory loss and paresthesia of the left lower limb. The episode lasted for 30 min. He decided to present himself to the emergency room. The neurological exam was normal. There was a difference of arterial pressure between the right arm (101/67 mmHg) and the left arm (127/78 mmHg). MRI of the brain revealed acute right PCA territory infarction (Fig. 1), with no visible arterial occlusion. CT angiography revealed the presence of a 24.1 mm thrombus in the post-stenotic aneurysm sac (Fig. 2).

**Fig. 3:**
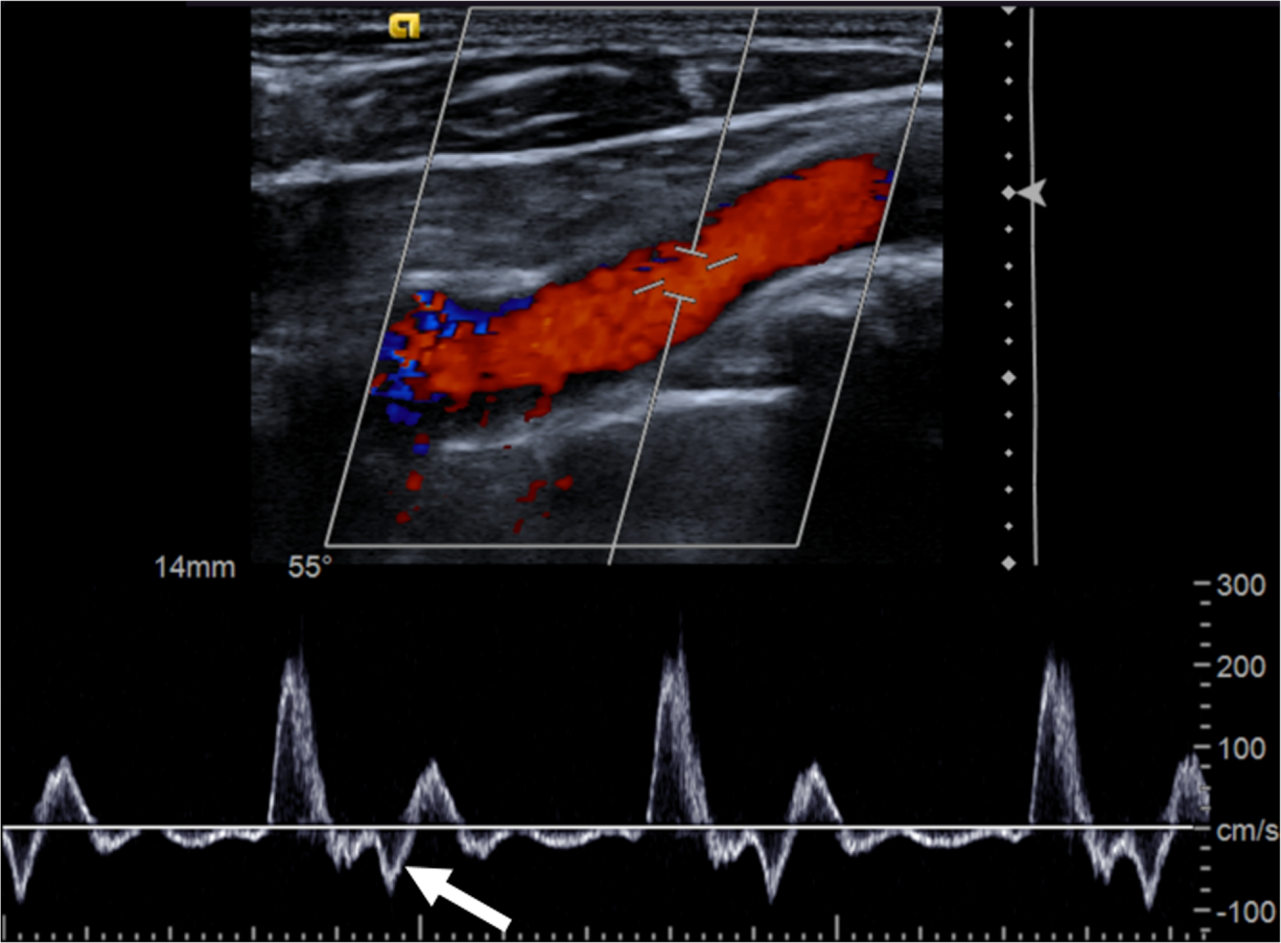
Doppler ultrasound of the right sub-clavian artery showing a retrograde flow (arrow)

An ultrasound examination of the supra-aortic arteries (Fig. 3) confirmed the post-stenotic dilatation and showed the presence of a small mobile thrombus on the distal wall the aneurysm, 5 cm away from the ostium of the right vertebral artery. Doppler shows retrograde reflux lasting about 0.45 s. During this reflux, the average maximum speed is − 12.8 cm / sec. The reflux can be therefore estimated to be 5.76 cm (12.8 × 0.45). The distance separating the edge of the mobile thrombus from the ostium of the right vertebral artery was 5 cm.

Blood cell counts and inflammatory markers were normal. Thrombophilia and vasculitis tests were negative. Electrocardiogram showed sinus rhythm with no arrhythmia during 1 week of telemetry. A trans-thoracic and trans-esophageal echocardiography was normal.

An enoxaparin treatment was started at curative dose. The thrombus disappeared after one week follow up. The patient underwent the surgery as planned in Sept 2017 with resection of the first rib, anterior scalenectomy and bridging of the aneurysm via a supra and sub-clavicular approach.

The patient always remained asymptomatic.

## Discussion and conclusion

The mechanism by which cerebral stroke is associated with arterial TOS has yet to be fully understood. This complication of arterial TOS has never been systematically studied, therefore its incidence is not known. We report the case of a patient whose doppler analysis shows that the retrograde flow embolism mechanism is both possible (amplitude of the reflux > distance between the aneurysm and the right vertebral artery ostium) and very likely (mobile thrombus in the post-stenotic aneurysmal sac).

Meumann et al. performed a review of 33 patients with ischemic stroke associated with arterial TOS [4]. A cervical rib was reported in 26 of these 33 patients. Median age in the review was 21 years, and the sex ratio 1/1. Twenty-one (81%) patients had preexisting symptoms evocative of arterial TOS, ranging from a few weeks to 12 years anterior to stroke. Nevertheless, only a minority of these patients had actually been diagnosed with TOS. Twenty (76%) patients presented with anterior circulation infarcts, four had posterior strokes, and two had infarcts from both circulations.

The first step of these embolic strokes is the SCA compression, intimal lesion leading to the formation of a thrombus. It is an acquired media disease secondary to extrinsic (bone compression) and intrinsic (turbulent / post-stenotic turbulent / non-linear flow) mechanical stress. The appearance of the thrombus can be explained by: intimal lesions or a low velocity flow in the bulge of the aneurysm sac.

Two mechanisms are proposed. First, a retrograde propagation of the thrombus to the vertebral or common carotid arteries. Vascular imaging has shown in some cases of arterial TOS associated strokes an extended thrombus in the brachiocephalic artery [9]. The second mechanism is a transient retrograde flow and embolism from the SCA post stenotic aneurysm to the vertebral artery. This mechanism has been highly suspected in three other cases [5, 8]. All three patients had a cervical rib. Retrograde blood flow was demonstrated in systole and diastole by Doppler ultrasound. One case affected the anterior circulation, two affected the posterior.

We cannot explain why 76% of patients had anterior circulation emboli in the above mentioned study [4]. Different factors must play a role in the probability of a thrombus entering preferentially the vertebral artery or the common carotid: localization of the embolic material, intensity and duration of the diastolic reflux, diameter of the vessel or the systolic flow velocity profile. Furthers detailed case studies are needed to understand if various mechanisms are involved in this clinical setting.

Our case, with an extensive ultrasound and doppler workup, advocates for a retrograde embolism. It is the first case to actually measure flux durations, distances and velocities, in order to prove that this mechanism is possible.

Although arterial TOS is the least common form of TOS, far behind neurogenic TOS, its complication can be the most devastating. Neurogenic TOS can result in chronic loss of neurologic function, venous TOS in local vein thrombosis. But arterial TOS can lead to limb ischemia or cerebral stroke. In our case, the patient had a good outcome.

This case raises two questions for our daily practice. Firstly, neurologists should always look for subclavian artery anomaly in a young patient stroke. The incidence of the etiology in all strokes is unknown and probably small, but should not remain undiagnosed to avoid stroke recurrence. A blood pressure difference of 20 mmHg between the upper extremities (significant but rarely found [17]), prior clinical symptoms suggestive of TOS and the presence of a cervical rib should raise suspicion.

Secondly, should vascular surgeons systematically introduce an anticoagulant treatment for every patient with arterial TOS, before surgery? Because the incidence of this complication is not known, it would be difficult to assess the benefice of a systematic treatment with known adverse effects.

## Data Availability

The data used and/or analyzed during the current study are available from the corresponding author on reasonable request.

## Abbreviations

CT: Computerized tomography
MRI: Magnetic resonance imaging
PCA: Posterior cerebral artery
SCA: Subclavian artery
TOS: Thoracic outlet syndrome
